# Supplementary material for: Morphological and Molecular Changes during Limb Regeneration of the Exopalaemon carinicauda
Source: Animals (Basel). 2024 Feb 22;14(5):685. doi: 10.3390/ani14050685 (PMC10931334; doi:10.3390/ani14050685)
Supplement: Supplementary file 1 [file animals-14-00685-s001.zip › FIgure legends.pdf]

Figure S1 GO classification of transcripts.

Figure S2 KEGG pathway classification of transcripts. The number above the bars indicates the number of transcripts annotated in different KEGG pathways. A). Cellular Processes; B). Environmental Information Processing; C). Genetic Information Processing; D). Metabolism; E). Organismal Systems.

Figure S3 NJ Phylogenetic tree analysis based on protein sequences, including *Procambarus clarkia* (XP_045616205.1), *Cherax quadricarinatus* (XP_053634753.1), *Homarus americanus* (XP_042205128.1), *Petrolisthes cinctipes* (KAK3885799.1), *Chionoecetes opilio* (KAG0710312.1), *Portunus trituberculatus* (XP_045116956.1), *Eriocheir sinensis* (XP_050715219.1), *Cancer borealis* (AFN25965.1), *Exopalaemon carinicauda*, *Penaeus chinensis* (XP_047502256.1), *Penaeus japonicus* (XP_042876315.1), *Penaeus vannamei* (XP_027207474.1), *Penaeus monodon* (XP_037790262.1), *Hyalella Azteca* (XP_018021806.1).

Table S1 Information on the primers used for real-time PCR.

Table S2 Summary statistics for sequencing data.
