# Supplementary figures and images for: Morphological and Molecular Changes during Limb Regeneration of the Exopalaemon carinicauda
Source: Animals (Basel). 2024 Feb 22;14(5):685. doi: 10.3390/ani14050685 (PMC10931334; doi:10.3390/ani14050685)

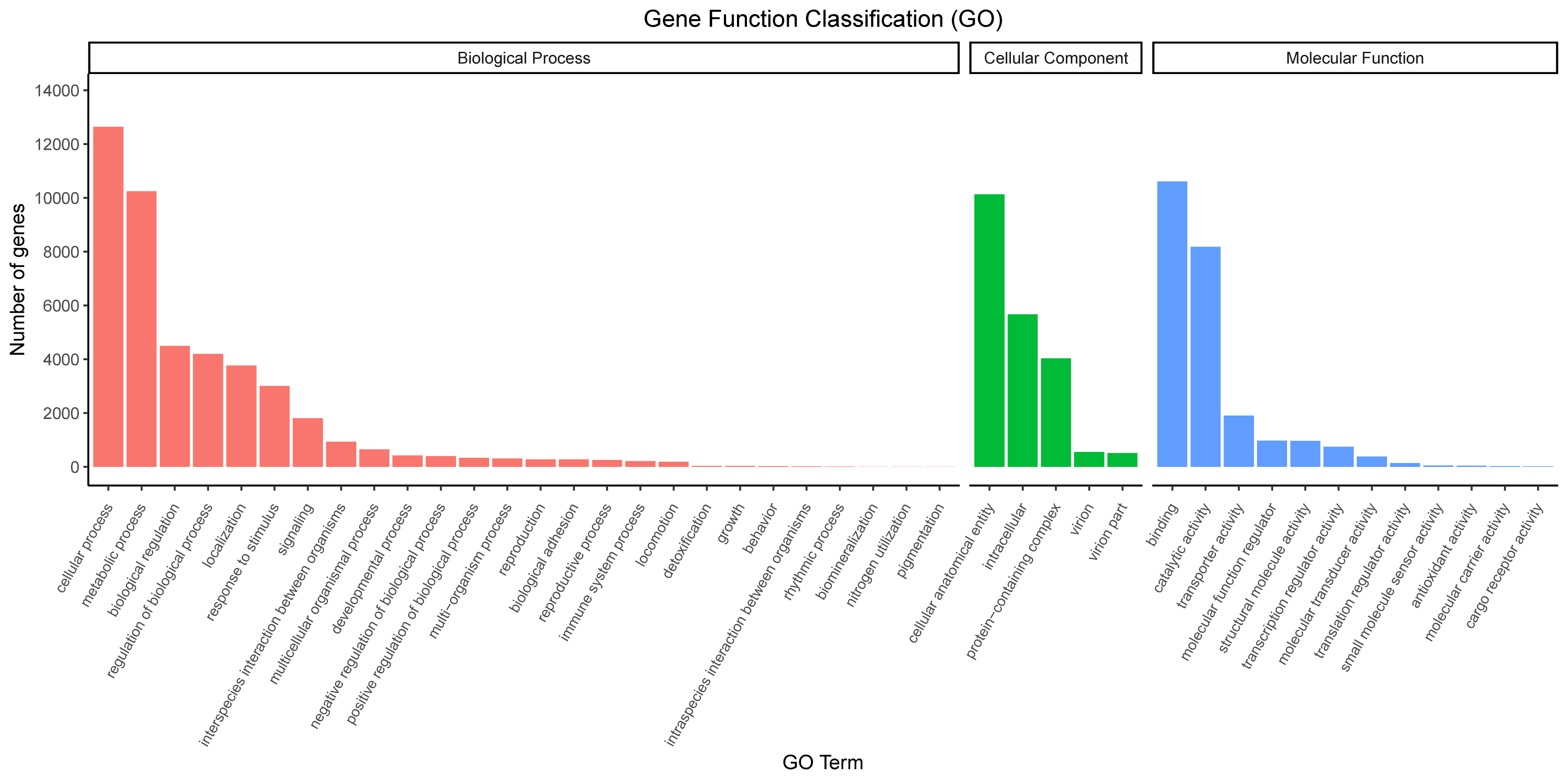

Supplement: Supplementary file 1 [file animals-14-00685-s001.zip › Figure S1.tif]

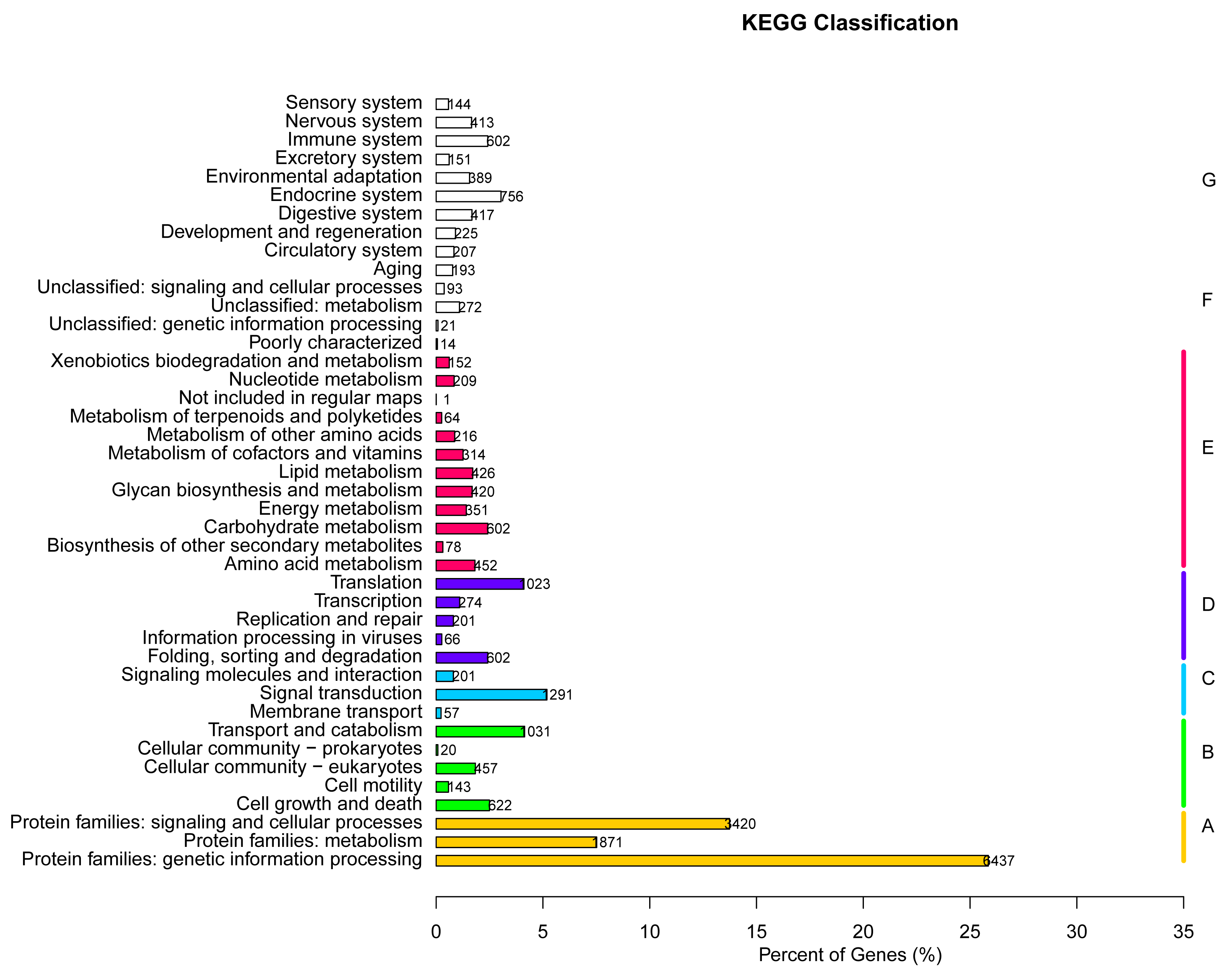

Supplement: Supplementary file 1 [file animals-14-00685-s001.zip › Figure S2.tif]

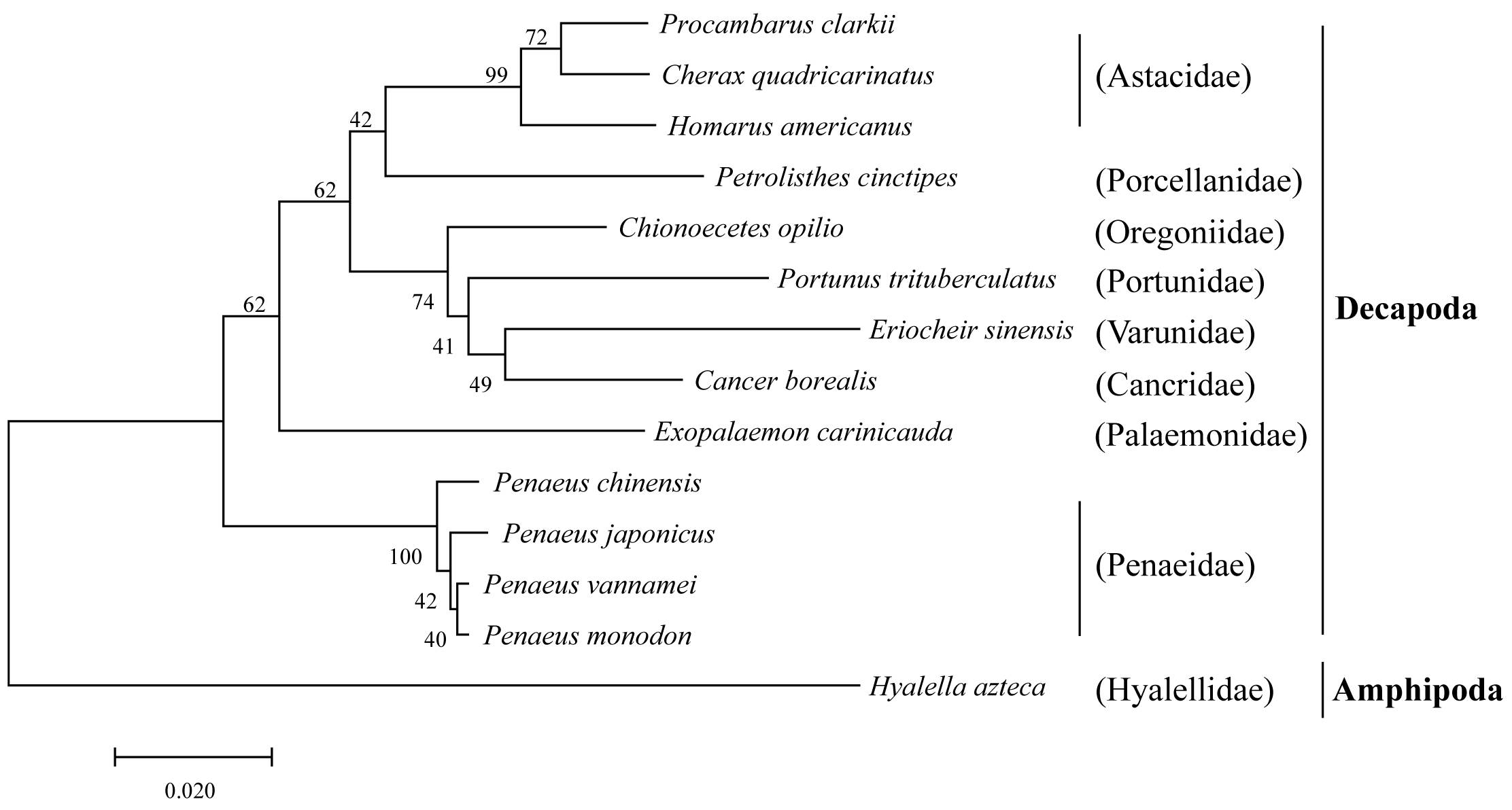

Supplement: Supplementary file 1 [file animals-14-00685-s001.zip › Figure S3.tif]
